# Estimated costs and benefits of participation in an extreme ritual in Mauritius

**DOI:** 10.1017/ehs.2025.10017

**Published:** 2025-08-22

**Authors:** Eva Kundtová Klocová, Radek Kundt, Pushkar Varma Puryag, Martin Lang

**Affiliations:** 1 LEVYNA: Laboratory for the Experimental Research of Religion, Masaryk University, Brno, Czechia; 2 University of Delhi, New Delhi, India

**Keywords:** costly signaling theory, cooperation, religion, ritual, Mauritius

## Abstract

Humans often participate in physically harmful and demanding rituals with no apparent material benefits. Although such behaviours have traditionally been explained using the lens of costly signalling theory, we question whether the canonical theory can be applied to the case of human cooperative signals and introduce a modification of this theory based on differential benefit estimation. We propose that along with cooperative benefits, committed members also believe in supernaturally induced benefits, which motivate participation in extreme rituals and stabilize their effects on cooperative assortment. Using Thaipusam Kavadi as a prototypical costly ritual, Tamil (ingroup) and Christian (outgroup) participants in Mauritius (*N* = 369) assessed the cost and benefits of Kavadi participation or hiking. We found that ingroup participants estimated material costs as larger than outgroups, physical costs as lower, and benefits as larger. These findings suggest that estimated costs may vary by modality and cultural expectations (e.g. Kavadi participants are not supposed to display pain), while supernaturally induced benefits were consistently reported as larger by ingroups compared to outgroups. We conclude that differential estimation of ritual benefits, not costs, are key to the persistence of extreme rituals and their function in the assortment of committed members, underscoring the role of differential estimation in the cognitive computation of signal utility.

## Graphical abstract

**Figure:**
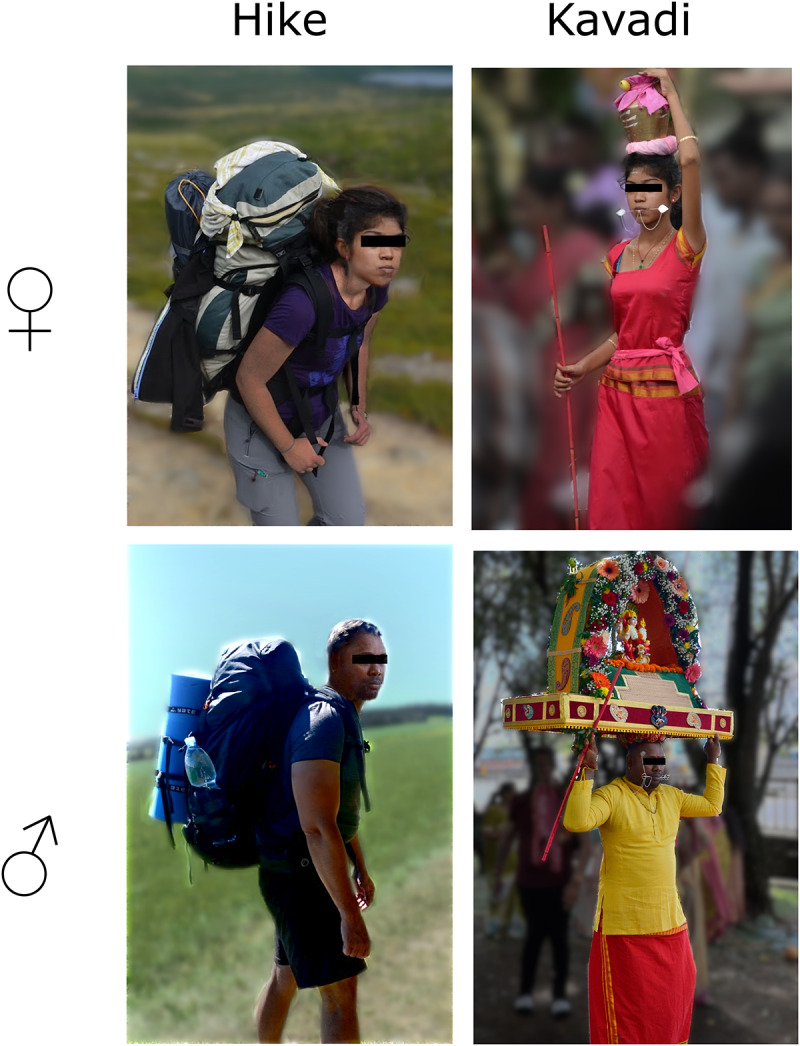

## Social media summary

Costly rituals endure because committed members see greater benefits – especially supernatural – despite high estimated cost.

## 1. Introduction

Humans, like other organisms, usually avoid activities that cause pain or waste energy. However, in many cultures, certain ritual practices deliberately involve physical harm and exertion, even when they offer no obvious benefits (at least from an outsider’s perspective). Examples of such rituals include skin piercing, scarification, tattooing, walking on burning coal, extreme dehydration, physical exhaustion, and other similar forms of suffering. Although puzzling at first sight, evolutionary scholars proposed that these behaviours might be explained using costly signalling theory (Barker et al., 2019; Bulbulia & Sosis, 2011; Cronk, 2005; Irons, 2001; Lang & Kundt, 2024; Power, 2017a; Sosis, 2003).

Originating in evolutionary biology (and with a counterpart in economics), costly signalling theory explains how organisms use costly signals to reliably advertise their hidden qualities (Spence, 1973; Veblen, 1899; Zahavi, 1975). The stability of the signal, that is, its functional persistence in the population despite the desire to manipulate the signal by low-quality individuals, is thought to be maintained by a tight association between the signal production capability and the underlying quality. In the most extreme case, a lack of certain qualities may even prohibit sending the signal, although most often both high- and low-quality individuals may produce the signal, but signal production is differentially costly (Grafen, 1990) or differentially beneficial (Lachmann et al., 2001) along the high–low continuum. In other words, the differential costs and benefits of signalling based on signaller quality lead to differential trade-offs that low- and high-quality signallers face (Számadó et al., 2022, 2023).

Although costly signalling theory has traditionally been used to explain mate selection or prey–predator interactions in non-human animals (Searcy & Nowicki, 2005), it has also received considerable attention in explaining human traits, such as a hunter’s quality or prosocial acts (Barclay et al., 2021; Bliege Bird & Power, 2015; Bliege Bird et al., 2001; Stibbard-Hawkes, 2019; Lang et al., 2024). Applying the theory to the specific case of ritual behaviour, it has been suggested that religious rituals and taboos can serve as signals of commitment to supernaturally sanctioned social norms (Alcorta & Sosis, 2005; Brusse, 2019; Bulbulia & Sosis, 2011; Chvaja & Řezníček, 2019; Iannaccone, 1994; Irons, 2001; Murray & Moore, 2009; Sosis, 2003; Uhrin & Bužeková, 2022). Participating in religious rituals is argued to stabilize intragroup collective action by reliably signalling cooperative intentions and commitment to joint interests. That is, rituals provide a platform for reliably sharing information about each member’s commitment and prosocial tendencies, protecting communities against free-riders (Lang & Kundt, 2024).

There is ample evidence for the association between participation in religious rituals and cooperative behaviour towards co-religionists. For example, participating in an extreme ritual in Mauritius was associated with increased contributions to the local temple organizing the ritual (compared to prayer; Xygalatas et al., 2013) and religious kibbutz members attending communal rituals contributed more in an economic game compared to secular kibbutz members (Sosis & Ruffle, 2003). Complementary to the link between religious practice and cooperativeness, the receivers of the religious signal (observers) also understand the signal to indicate cooperative intentions: there is evidence that people perceive religious signallers as more trustworthy (Hall et al., 2015; Shaver et al., 2018; Soler, 2012), as more suitable potential husbands (Xygalatas et al., 2022), and this effect is stronger in religious individuals, who trust religious signallers more than secular individuals trust secular signallers (Chvaja et al., 2023). Moreover, religious practice is also positively associated with the probability of being nominated to be part of one’s social supportive network in Tibet (Ge et al., 2024), India (Power, 2017b), and the USA (Chvaja et al., 2025).

Providing further support for the link between religious signalling and cooperation, there is evidence that religious costly signals may be adaptive in the long term. Using ethnographic data on the costliness of rituals and religious practices in small-scale societies, it has been shown that signal costliness scale with the external (environmental and social) pressures on intragroup cooperation such as warfare or food availability (Learmouth et al., 2024; Sosis et al., 2007; Sosis & Bressler, 2003; c.f., Šaffa et al., 2022). In these contexts, engaging in physically taxing or painful rituals can function as credible signals of group commitment and trustworthiness. By filtering out less dedicated individuals and reinforcing group solidarity, such costly practices help stabilize cooperation and collective action over time, thus enhancing group survival and individual fitness within the group.

However, given the evidence for the functional assortment of cooperative individuals facilitated by participation in religious rituals, it is puzzling why free-riders would not participate in such rituals and then exploit the cooperative efforts of other participants. Although repetitive interactions and associated reciprocity may go towards explaining part of this effect (Lang et al., 2022), the canonical costly signalling model does not assume repeated interactions. For instance, in the predator–prey dynamic, the signalling encounter is usually a singular event independent of previous encounters (Caro, 1994). Crucially, unlike most non-human animal signalling, human signalling of cooperative intentions can be independent of the underlying quality – in this case, even free-riders can send the costly signal (e.g. participate in a religious ritual) and reap its benefits. It follows that the signalling systems should be unstable in this case because they could be undermined by manipulators, seemingly contradicting the empirical evidence listed above (c.f. Chvaja, 2025).

Recognizing this problem more than two decades ago, Sosis (2003) suggested that ardent members of religious groups estimate the costs of ritual participation as lower than outsiders, effectively modifying the cost/benefit trade-off of signalling that individuals compute when deciding whether to signal (differential cost estimation). Because religious individuals often partake in the rituals of their community since childhood, they do not estimate the participation to be so costly due to habituation to their performance. For ardent members, participation is normalized and can be easily performed with few obstacles, unlike for newcomers who are not aware of all the ritual nuances and face a steep learning curve. Nonetheless, although crucial for the application of costly signalling theory on ritual, this proposition has rarely been tested. For example, in his ethnographic work in Greece, Xygalatas (2012) found that participation in a fire-walking ritual is often referred to as easy and non-threatening among committed ritual performers, and a recent experimental study in Mauritius showed the same relationship with a Hindu extreme ritual (Demiroglu & Xygalatas, under review). On the other hand, experiments conducted in Spain (Chvaja et al., 2023) and the USA (Lang et al., 2025) showed that Christian participants estimate the religious signal to be more costly than secular participants, an opposite effect than should be expected.

To explain these disparate results, we propose that religious commitment signals are stabilized by the interplay between two different types of benefits that potential performers estimate. First, the cooperative benefits stemming from the signalling endeavour indicating commitment to norms regulating collective action, and second, the supernaturally induced benefits that believers hope to obtain by performing the signalling action – ritual – itself. Although the former benefits are recognized in- and outside the religious group by observing the cooperative output of that group, the latter benefits are not necessarily related to cooperative benefits and would be recognized (i.e. believed in) only by the committed members. Combining these benefits, ardent members of religious groups would be motivated to perform the rituals for the supernaturally induced benefits (e.g. miraculous healing, god-send business success or protection, salvation and blessing) while simultaneously signalling commitment to the particular religious system.

The perception of such supernaturally induced benefits has two intertwined effects stabilizing commitment religious signals. On the one hand, dedicated members are motivated to increase the cost of ritual participation to increase the supernaturally induced benefits. On the other hand, this increased cost deters free-riders whose cost/benefit trade-off is negative because they include in their cognitive computations only the cooperative/free-riding benefits while discarding the other supernaturally induced benefits. Such a complex link between supernatural benefits, costly rituals, and cooperation makes religious traditions unique cultural systems, which may explain their evolutionary success (Lang & Kundt, 2020; Purzycki & Sosis, 2022).

To test this cost-as-supernatural-benefits model, we conducted a preregistered study in Mauritius where we asked Tamil Hindus (ingroup) and Christian (outgroup) participants to imagine either participation in a Thaipusam Kavadi – an intense Hindu ritual – or in an effortful hike (control condition). We obtained the estimated costs and benefits of such participation and investigated whether the estimation of these costs and benefits would differ between in- and outgroups. We put forward the following hypotheses:

- H1a. According to the differential cost estimation model proposed by Sosis (2003), ingroup potential signallers would estimate the cost of the ritual as lower compared to outgroup non-signallers, and this difference would be larger in the ritual compared to the control condition.

- H1b. According to the proposed cost-as-supernatural-benefits model, ingroup potential signallers would estimate the cost of the ritual as larger compared to outgroup non-signallers, and this difference would be larger in the ritual compared to the control condition.

- H2. The number of listed benefits of ritual participation will be larger in the ingroup potential signallers compared to the outgroup non-signallers, and this difference will be larger in the ritual compared to the control condition.

- H3. Ritual will be rated as more efficacious in securing supernaturally induced benefits (both material and spiritual) by ingroup potential signallers compared to outgroup non-signallers, and this difference will be larger in the ritual compared to the control condition.

- H4. Increased ritual intensity will be associated with larger increased reported benefits in ingroup potential signallers compared to outgroup non-signallers, and this difference will be larger in the ritual compared to the control condition.

## 2. Methods

### 2.1. Field site

Mauritius, a small island nation, is located in the south-western Indian Ocean, approximately 2000 km (1200 miles) off the south-eastern coast of Africa. It lies just north of the Tropic of Capricorn and is part of the Mascarene Islands, along with Réunion and Rodrigues. Its settlement occurred in several waves during the modern period, and its highly diverse demographic composition reflects the colonial and migratory history of the area. European colonizers, mainly the French, gradually brought slaves to the island, mostly from Africa and Madagascar. Subsequently, under the British administration, indentured labourers were brought to the island from areas of India and Pakistan. People of Indian origin make up a slight majority of the population, closely followed by people of African origin, and then by minority groups of Sino-Mauritians and Franco-Mauritians.

Religious affiliation still closely follows ethnic origins, although there are changes due to the proselytization of some religious groups, but also due to increasing secularization. The largest ethnic group – Indo-Mauritians – are primarily Hindu and are divided into several specific subgroups according to their area of origin. In addition to the general Hindu group, there are groups of Tamils, Telegu, and Marathi, who differ in their emphasis of veneration of different gods from the Hindu pantheon. There are also Indo-Mauritian Muslims, mostly belonging to Sunni Islam, although they are a minority among Indo-Mauritians. Afro-Mauritanians adhere mainly to Christianity, especially Catholicism, but there are also many Protestant groups on the island. A minority group of Mauritians of French origin also adhere to Christianity. There are also Buddhist groups on the island (especially among Sino-Mauritians). Alongside this religious pluralism, there is great religious freedom on the island, with the state actively supporting most of the major religious traditions.

As a stimulus for our study, we used Thaipusam Kavadi, which is the central ritual of several Tamil Hindu festivals in Mauritius celebrated in January or February. The ritual follows 10 days of preparation involving vegetarian fasting, abstinence, prayers, and decorating *kavadi* – portable altars adorned with offerings. These structures, often weighing over 50 kg, are carried or pulled by devotees, sometimes using skin hooks. Piercings are integral to this ritual, ranging from small hooks to spikes and rods, with some devotees almost fully covering their bodies (Xygalatas et al., 2021). The hours-long procession includes trance-like dancing and, in some locations, climbing temple steps to complete the offering. People participate in this ritual for many different reasons (such as healing a chronic condition), often influenced by their individual life situation and socioeconomic status (Xygalatas & Maňo, 2024).

Children are often part of the procession, either carried by parents or walking with the procession. Some children of relatively young age (e.g. from 7/8 years old) also actively participate in the procession by carrying a (small) kavadi and/or having a piercing. An annual participation in the Thaipusam Kavadi is not necessarily expected, and many participate occasionally when making a vow to Murugan to obtain help with an issue or are motivated by gratitude to Murugan for solving past problems. Nevertheless, normative expectations might differ based on socioeconomic status because leaders of the local community might be expected to participate and lead the procession (Xygalatas et al., 2021). Note that while this ritual is predominantly performed by Indo-Mauritians, there are occasionally some Afro-Mauritian participants (often from mixed marriages). We control for this possibility in our sampling strategy (see below), yet in the Christian community, it is not an expected behaviour.

Our data collection locations encompassed various locations across the island, including parks and streets in different cities, shopping centres, transit stops, and other public spaces where people typically gather and have spare time. Data collection focused on engaging individuals in these diverse, high-traffic areas. To increase the likelihood of obtaining sufficient respondents from the selected groups, we also targeted areas known to be populated by Tamils or Christians. Data were collected by Mauritian research assistants (all female, 5 Hindu, 3 Muslim, 1 Tamil).

### 2.2. Participants

We originally aimed to recruit 300 participants and performed an associated power analysis using the package *simr* (Green & Macleod, 2016). This power analysis revealed that this sample size would allow us to detect the following interaction effects with 80% probability (i.e. the difference between outgroup versus ingroup participants in the slopes going from the control to ritual condition): 0.8 for H1 and H3 assuming normal distribution (i.e. difference in slopes of 0.8 on a 5-point Likert scale), 0.43 on a log scale for H2 assuming a Poisson distribution (i.e. difference of 1.3 mentioned benefits), and 0.75 for H4 assuming normal distribution (i.e. difference in slopes of 0.75 on a 4-point Likert scale). Note that we used power estimation for gaussian models (H1, H3, H4) as an approximation of the needed sample size although we planned to use cumulative link models in our analysis. Despite the original expectations affected by the unpredictability of data collection in the field, we recruited 403 participants, 369 of whom finished the study (180 women; M_age_ = 37.5, SD_age_ = 14.4, range = 18–74), increasing the probability that we would detect the prespecified effects or detect even smaller effects. Our final sample comprised 96 ingroup and 89 outgroup participants in the ritual condition, and 73 ingroup and 111 outgroup participants in the control condition. Assignment to the ritual and control condition was random based on Qualtrics algorithms.

Data were collected using a survey built through the Qualtrics app. The survey was read aloud by a research assistant in Mauritian Creole and shown to respondents, when required, from a tablet. For ease of processing, the research assistants wrote the answers to the free-list question on a paper marked with an ID, which was then linked to the answers entered on the tablet. There was no monetary reward for participation. After data collection, we excluded eight participants based on predefined criteria: one ingroup participant who would never consider taking part in Kavadi and seven outgroup participants who took part in Kavadi. Note that although we preregistered these exclusion criteria, we also provide a reanalysis of our main hypotheses including these participants in the Supplementary Material, section S4, to show the impact of this exclusion (there is none).

### 2.3. Design

The study was designed as a 2 × 2 between-subjects experiment, with the first factor related to religious affiliation (not manipulated) and the second factor related to the nature of the stimulus (manipulated). As affiliation to a specific religious tradition and ethnicity are tightly associated in Mauritius (Shaver et al., 2018), we divided our participants for the first factor – group – into in-group potential signallers (Indo-Mauritian ethnicity, predominantly from Tamil communities that perform Kavadi rituals) and outgroup non-signallers (Afro-Mauritian ethnicity, predominantly Christians, individuals rarely take part in Kavadi rituals). The second factor – treatment – refers to a random assignment of participants either to a ritual or control condition. In both conditions, participants were presented with a photo of another person engaging in an activity and asked to imagine that they would participate in the same way in this activity. In the ritual condition, participants estimated the costs and benefits of participation in the Kavadi ritual. In the control condition, participants estimated the costs and benefits of participation in a comparable effortful non-ritual activity: hike with a heavy backpack.

The motivation for choosing these comparisons was to first assess whether outgroups, who do not regularly participate in the ritual, differentially assess the cost and benefits of participation. Indeed, Christians rather do not participate in the ritual, despite the ritual being a public procession in cities where Christians live. The comparison of Kavadi participation with hiking aimed to control for the possibility that ingroups assess the costs and benefits of a demanding physical activity differently than outgroups. Beyond this main control, hiking might also control for other important benefits that the in-/outgroup participants might perceive differently, including improved health (Xygalatas et al., 2019) or signalling self-control (Chvaja et al., 2023).

**Figure 1.**
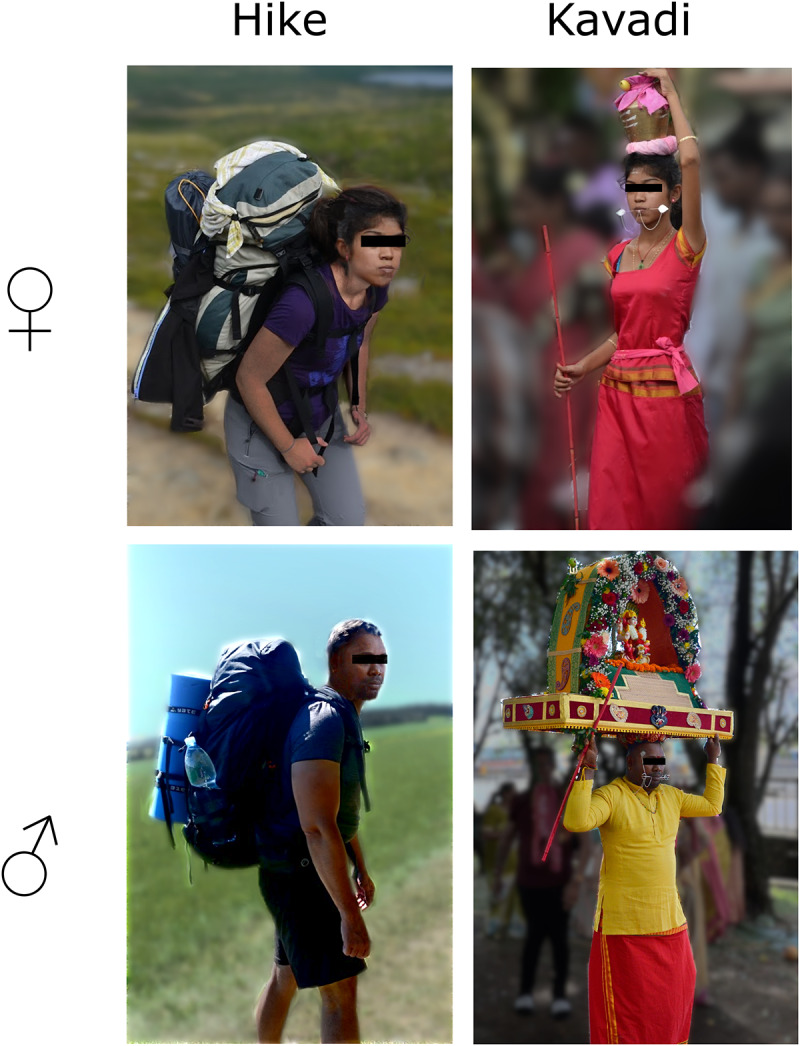
Visual stimuli, systematically varying along two dimensions: activity type (hike versus kavadi) and participant gender (women ♀ versus men ♂).

Importantly, the stimuli differed for men and women because the form of participation in Thaipusam Kavadi is highly gendered (women usually engage in less-intense activities during ritual procession). Specifically, men in the religious condition saw a picture of a male Kavadi performer with several piercings and carrying a kavadi, whereas women saw a woman with pierced cheeks and a pot on her head. In the control condition, men saw a man with a heavy backpack in the mountains, and women saw a woman with a similar backpack (see Figure 1 for the visual stimuli).

### 2.4. Measures

**Figure 2.**
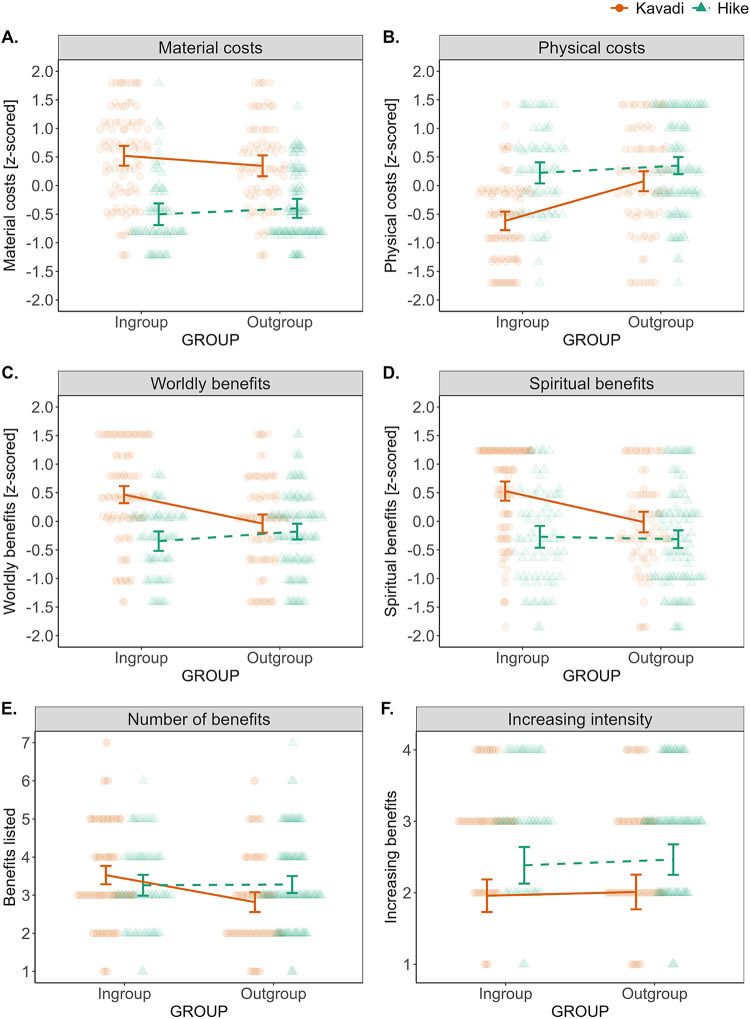
**Overview of results of the main hypotheses tests.** Error bars are 95% CIs and scatter plots represent raw data. Note that for the sake of brevity, we plot the results collapsed along the originally expected categories: material/physical costs and spiritual/worldly benefits. **(A)** Ingroup participants estimate Kavadi participation to be more materially costly (time + finances) than outgroup participants, and this increase is larger compared to hiking. **(B)** Ingroup participants estimate Kavadi participation to be less painful and effortful than outgroups, and this decrease is larger compared to hiking. **(C,D)** Ingroup participants estimate Kavadi participation to be more likely to bring both worldly and spiritual benefits compared to outgroups, and this increase is larger compared to hiking. **(E)** Ingroup participants listed larger number of benefits for Kavadi participation compared to outgroups, and this difference was larger compared to hiking. **(F)** The effect of increasing Kavadi intensity does not differ between in- and outgroups.

To test H1, we designed four questions using 5-point Likert scales. Specifically, we asked participants to estimate the time it would take them to prepare for participation in the kavadi/hike, the financial cost of kavadi/hike participation, and about the pain experienced and needed effort during the kavadi/hike. When designing these measures, we planned to tap into a latent variable with two different facets: material (time and finances) and physical (pain and effort) costs. Our expectation that costs would be of two different kinds was supported in an exploratory factor analysis with oblimin rotation, where the one-factor solution revealed a poor fit to the empirical data (standardized Cronbach’s alpha = 0.23). As preregistered, we expected that participants might be differently motivated to answer questions about material and physical costs and indeed, these two different cost types were negatively correlated (Pearson’s *r* = −0.118, *p* < 0.001). However, even when we created composite variables separately for material and physical costs, the split-half reliability calculated using the Spearman–Brown prediction formula for the material (0.54) and physical (0.73) cost did not show high internal consistency. Thus, we decided to report these items separately, although we plot the results in Fig. 2 along these originally planned analyses.

To obtain the main variable for testing H2, we employed the free-listing method (Purzycki, 2025) and asked participants to list up to 10 benefits that the activities they saw (kavadi/hike) may entail. The number of benefits listed served as our main outcome variable in H2. In addition, we also used the free-listing method for its original purpose, that is, to gain a richer understanding of the benefits respondents believed each presented activity brings. Data from free-lists were coded by two independent coders (see Supplementary Material for inter-rater reliability) and analysed using the AnthroTools package (Purzycki & Jamieson-Lane, 2017) to determine the salience of benefit categories.

Regarding the likelihood that ritual/hike participation would bring specific benefits (H3), we used again a 5-point Likert scale (Unlikely to Certainly) and asked about the likelihood of four benefits: healing from a serious illness, helping with financial troubles, purifying oneself, and getting rid of evil spirits. Note that this question followed the free-list prompt about activity benefits, such that our suggestions would not affect the number of benefits listed, that is, the test of H2. Again, in selecting these benefits, we assumed one factor with two facets that could be described as worldly benefits (health, money) and spiritual benefits (purification, getting rid of evil). Exploring whether the one-factor solution would be sufficient (using factor analysis with oblimin rotation), we observed that all four items were intercorrelated (χ^2^ (6) = 362.90, *p* < .001) with adequate sampling (MSA = 0.71), factor loadings (ranging between 0.53 and 0.84), and sufficient coherence (Cronbach’s α = 0.72). Thus, we averaged the *z*-scores of all four variables to create the composite variable of perceived likelihood of benefits.

Finally, we also asked about whether increasing the intensity of kavadi/hike participation would increase the likelihood of the benefits (H4; 4-point Likert scale; Not at all to Certainly). This question was motivated by asking directly about the association between ritual costs and benefits.

We further asked two questions regarding whether participants think taking part in ritual/hike would increase their trustworthiness (5-point Likert scale; Unlikely to Certainly) and whether increasing participation intensity would also increase the effect on trustworthiness (4-point Likert scale; Not at all to Certainly). The former question assessed whether participants include cooperative benefits of kavadi/hike participation directly in their cost/benefit analysis when asked explicitly and whether increasing costs translates into increasing benefits.

Participants’ religious affiliation served as a factor in our interaction models, dividing the participant pool into ingroups (Tamil participants – kavadi ritual is practised in the Tamil community) and outgroups (Christians who usually do not take part in the Tamil kavadi ritual). If participants followed both Tamil and Christian practices (e.g. in a mixed marriage), we counted them as Tamil as long as they participated in kavadi or helped with its preparation before (three participants counted as Tamil, four as Christians). To assess religiosity as a more nuanced predictor of estimated costs and benefits within Tamil participants, we asked four questions: frequency of ritual participation, frequency of prayer, importance of religion in everyday life, and religiosity compared to other people participants know. The religiosity scale showed moderate adequacy (MSA: 0.7, Cronbach’s alpha = 0.72), and we standardized each measure as *z*-scores and averaged those measures to arrive at the latent variable of religiosity.

Covariates that we planned to use to adjust our statistical models included the number of times participants already took part in the kavadi ritual directly, whether they participated at least indirectly (walking procession, helping prepare kavadi, helping a friend), and if not, whether they would consider such a participation. Next, we asked about age, gender, and socioeconomic status assessed by four measures: occupation (three independent local coders rated job prestige), education level, and house and car ownership. Finally, to check whether our task was meaningful, we asked participants at the end of the survey how difficult it was to imagine participating in kavadi/hike (5-point Likert scale; Not difficult at all to Impossible).

### 2.5. Analysis

Analyses were conducted in R (R Core Team, 2020) using the *glmmTMB* package (Brooks et al., 2017) and *ordinal* package (Christensen, 2018). As preregistered, our general model structure involved an interaction between group (Ingroup versus Outgroup) and treatment (Ritual versus Control), and a varying intercept term for gender because the stimuli differed for women and men. In terms of the data-generation processes, whenever we analysed a composite variable, we assumed a gaussian distribution rising from scores on discrete, bounded variables – Likert scales. When we analysed single Likert-scale items, we used a cumulative link model that estimates the probabilities of crossing from one ordered level of the outcome variable to a higher level. However, because the ordinal package allows for random intercepts only for factors with three or more levels, we included gender in these models as a fixed factor. For count data, we preregistered using either Poisson or a negative binomial model (in the case of overdispersion in the Poisson model), yet none of the models fitted the data well. Instead, we found that ordinary least square regression fitted the data best (the data were roughly normally distributed), and we used this model instead.

In terms of our modelling strategy, we first fit the basic interaction models and in the second step, we adjusted these models for potential covariates, namely age (older participants may estimate some of the costs as larger), socioeconomic status (higher SES participants often engage in less-intense Kavadi performance), and experience with Kavadi (more experienced participants might have a more realistic estimate of participation costs). In the third step, we examined the effects of religiosity on the cost/benefit estimation within the ingroup. Finally, we checked whether the effects differ between women and men. We report the main hypotheses tests in the main text (the first analytical step) and the remaining analyses testing the robustness of our findings are presented in Supplementary Material, section 4 (the results reported in the main text withstand the robustness checks).

## 3. Results

As a manipulation check, we fitted a cumulative link model asking participants how difficult it was to imagine participating in the portrayed activity. The cumulative link model revealed no reliably estimated difference between the ritual and hike conditions (β_[kavadi vs hiking]_ = −0.25, 95% CIs = [−0.64 to 0.14]), suggesting that both conditions were valid for participants. Yet, we observed that it was more difficult for outgroups to imagine participating in Kavadi compared to ingroups (β_[in vs out]_ = 2.52, 95% CIs = [1.90 to 3.16]), and that this difference was smaller in the hike condition (β_interaction_ = −2.32, 95% CIs = [−3.15 to −1.50]). The model predicted that the most probable response (55%) for ingroups was that it was ‘not difficult at all’ to imagine kavadi participation. In contrast, for outgroups, the most probable answer (41%) was ‘very difficult’. This result indicates that outgroup participants have low experience with taking part in Kavadi, presenting a valid simulation of the situation where potential outgroups may want to partake in a ritual and need to estimate its costs and benefits. It is the in-/outgroup difference in this estimation that we are interested in the current study.

### 3.1. Hypothesis 1 – estimated costs

Using cumulative link models for the separate cost items, we did not find a well-estimated difference between ingroups and outgroups for time spent preparing for Kavadi (β_[in vs out]_ = 0.33, 95% CIs = [−0.20 to 0.86]), and although ingroups estimated the Kavadi preparation to be more time-consuming than preparation for hiking (β_[kavadi vs hiking]_ = −2.45, 95% CIs = [−3.08 to −1.84]), this difference was similar in outgroups (β_interaction_ = 0.31, 95% CIs = [−0.48 to 1.10]). Estimated costs were much more salient in the financial domain: outgroup participants judged the ritual preparation as less costly compared to ingroups (β_[in vs out]_ = −1.005, 95% CIs = [−1.56 to −0.45]), and this difference was practically zero in the hiking condition (β_interaction_ = 0.99, 95% CIs = [0.23 to 1.7]). For instance, the joint probability of saying that Kavadi preparation cost more than 4000 MUR (options 3, 4, and 5) was 76% for ingroups but only 54% for outgroups. To underscore this point, several ingroup participants also mentioned that our financial cost scale was insufficient, and that participation would cost more than they could select. See Fig. 2A.

When preregistering H1, we also preregistered a caveat that material and physical costs may have different estimation biases due to the cultural norm about Tamil Hindus not showing pain during Kavadi. Indeed, outgroup participants judged Kavadi as more painful compared to ingroups (β_[in vs out]_ = 1.60, 95% CIs = [1.03 to 2.18]), and this difference was smaller in the hiking condition (β_interaction_ = −1.30, 95% CIs = [−2.07 to −0.53]). The probability of selecting an option that the Kavadi would not hurt or hurt only a little was 77% for ingroups but 41% for outgroups. The same pattern of results was found for estimated effort: Kavadi was perceived as more effortful by outgroups compared to ingroups (β_[in vs out]_ = 1.16, 95% CIs = [0.61 to 1.72]), and this in- versus outgroup difference was smaller in the hiking condition (β_interaction_ = −0.92, 95% CIs = [−1.69 to −0.17]). See Fig. 2B.

Together, these results show that although financial costs tend to be overestimated by ingroups, physical costs are underestimated. These results hold when we adjust our models for control variables (see Supplementary Material, Table S2).

### 3.2. Hypothesis 2 – the number of listed benefits

**Figure 3.**
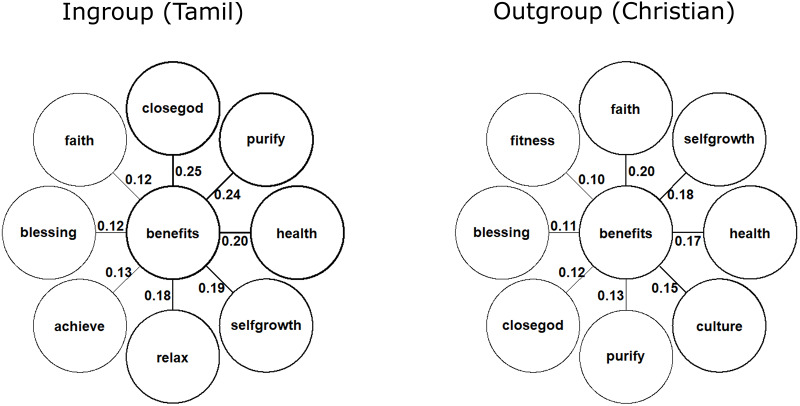
**Flower plots display the most salient benefits of Kavadi participation for ingroup and outgroup members.** The top outer circle shows the most salient item for each group, with descending items listed clockwise. Numbers and weighted border lines indicate the relative salience of each item (Smith’s S).

Analysing the number of benefits people listed in the free-list task, we found that outgroup participants listed less benefits for Kavadi participation compared to ingroup participants (β_[in vs out]_ = −0.71, 95% CIs = [−1.06 to −0.36]), and this in- versus outgroup difference was practically zero in the hiking condition (β_interaction_ = 0.73, 95% CIs = [0.23 to 1.23]). We also planned to harness the strength of free-list data, namely understanding the content of specific benefits of Kavadi participation. The salience analysis reported in Figure 3 revealed that for ingroups, the most salient benefit was experiencing closeness to God, often described as feelings of transcendence or a strong positive emotional connection. The second benefit that came up with a similar salience was purification, highlighting its importance for spiritual life of Tamils. In contrast, outgroups identified the most salient benefit as strengthening or developing one’s faith, emphasizing commitment to a religious tradition, and the expansion of a trait or attitude rather than an experience. This result is further supported by frequent mentions of reinforcement of cultural identity through the ritual. The benefits listed most often by the ingroup thus refer more to something that needs to be felt, or otherwise experienced, with an external source. Outgroups point rather to identity and personal change coming from within. Interestingly, ingroups also reported relaxation as a benefit, primarily related to stress reduction and peace of mind, despite the ritual’s physical demands.

### 3.3. Hypothesis 3 – estimated probability of benefits

Using a composite variable of the estimated probability of benefits (averaged *z*-scores of four items), we found that outgroups perceived Kavadi participation to be less likely to help with illness, finance problems, purity, and getting rid of evil spirits than ingroups (β_[in vs out]_ = −0.52, 95% CIs = [−0.72 to −0.33]), and there was no difference between outgroups and ingroups in the hiking condition (β_interaction_ = 0.59, 95% CIs = [0.31 to 0.86]). This result holds when replicated using cumulative link models for all the individual items, and, thus, applies to both worldly (illness, finance) and spiritual (purity, getting rid of evil) benefits. For example, the modal answer to the question of whether participating in the Kavadi would help with financial issues was ‘certainly’ (5 of 5) in ingroups but ‘quite likely’ (3 of 5) in outgroups. See Fig. 2C,D.

### 3.4. Hypothesis 4 – increasing probability of benefits with increasing costs

Our final hypothesis pertained to directly testing the idea that increasing costs are related to increasing benefits. Using the cumulative link model, we found no reliably estimated difference between ingroup and outgroup participants in judging the increased effectiveness of more intense participation in the Kavadi ritual (β_[in vs out]_ = 0.25, 95% CIs = [−0.32 to 0.81]) and the same was true for the hike condition (β_interaction_ = −0.16, 95% CIs = [−0.94 to 0.61]). Looking at modal answers, for both ingroups and outgroups, the modal answer was ‘Not at all, increasing intensity would not make the advantages more likely’.

### 3.5. Exploratory test – perception of cooperative benefits

Finally, although our theory does not necessarily assume that participants would directly reflect on cooperative benefits that Kavadi participation potentially bestows on them in the Tamil community, we asked directly about the perception of such benefits. We found that outgroup participants reported that Kavadi participation would increase their trustworthiness less than ingroup participants, but this effect was poorly estimated (β_[in vs out]_ = −0.37, 95% CIs = [−0.90 to 0.16]) and, subsequently, so was the slope difference compared to the hike condition (β_interaction_ = 0.54, 95% CIs = [−0.21 to 1.30]). However, the modal answer for ingroups was ‘a little bit likely’ that people will trust them more while for outgroups the modal answer was ‘unlikely’.

## 4. Discussion

We investigated whether ingroup participants of Tamil ethnic and religious identity would differ in their perception of the costs and benefits of participation in their community’s important ritual (Thaipusam Kavadi) from outgroups (Christians of Afro-Mauritian ancestry). As a control, we also assessed the perception of these two participant groups of the costs and benefits of hiking, which was visually matched to the intensity of ritual participation. The results revealed that ingroups estimated financial costs of ritual participation to be larger, physical costs to be smaller, and benefits to be larger compared to outgroups. Importantly, these differences were larger in the ritual compared to the hike condition. We discuss the relevance of these findings for the broader framework of costly signalling theory of religion and situate some of the unexpected findings into the local context of the Tamil religious community in Mauritius.

Previous theories argued that because accustomed ritual participants are well-versed in the performance (usually from early childhood), the performance is relatively easier compared to novices/outsiders who might attempt ritual participation for the first time (Lang et al., 2022; Sosis, 2003). Indeed, Tamil children are socialized relatively early into the performance of this ritual (either as active participants or as walking with the procession), and it is likely that they are also socialized into the benefits that such performance bestows. As the costs are thus perceived as lower for the hardened members who should have cooperative intentions towards other group members, ritual performance should facilitate collective action. Moreover, whenever the group needs to protect their resources more, the ritual costs increase so that only committed members who perceive the costs as lower are still willing to undertake the trade-off where the benefits did not change (Sosis & Ruffle, 2003; Xygalatas et al., 2013).

In line with this theoretical prediction, we observed that ingroups estimated the physical costs of ritual participation to be lower compared to the estimation of the outgroups (similarly as in (Demiroglu & Xygalatas, under review). However, it is crucial to interpret this result within the specific context of Thaipusam Kavadi, where showing pain is an indication of insincere motivations or even an unworthy state of the participant (similarly as dropping the pot with milk women carry on their head during the procession). That is, even though ritual participation is supposed to be difficult, committed members should handle this difficulty without (showing) any pain or showing of effort, a pattern also observed in fire-walking rituals in Greece (Xygalatas, 2012).

However, our ethnographic observations suggest that although people indeed try to withstand the pain during piercing with a calm face, there are often signs of suffering, sometimes ostensibly visible in younger ritual participants. It is possible that our results on lower estimated pain and effort reflect the strong cultural consensus on not showing pain (inaccessible knowledge to outgroups), rather than the actual feelings one has during participation. As a matter of fact, we believe that if not asking about pain but, for example, the weight of the kavadi that one carries, ingroups would be inclined to overestimate this number as well (but would still downplay the pain). Our previous research in the context of US and European Christian populations showed that committed members tend to report the ritual activity as more effortful (Chvaja et al., 2023; Lang et al., 2025).

Even more problematic for the argument that commitment decreases the perceived cost of ritual participation are our results of financial costs, where ingroups estimated these costs to be larger compared to outgroups. Congruent with our explanation of cultural expectations, financial costs are not expected to be downplayed. Moreover, outgroups may not be privy to all the different investments that Kavadi participation requires, which should, in theory, push them towards participation to obtain/free-ride on the cooperative benefits of committed members. For example, they may not be aware of all the ritual paraphernalia that need to be bought, the types of materials that can be used to build the kavadi, transportation of the kavadi or buying the piercings that performers sport during the procession. Why would the ritual not be performed by outsiders to obtain the cooperative benefits of ritual-facilitated assortment?

We believe that the key difference lies in the estimated benefits of ritual participation. Indeed, although ingroups perceive Kavadi participation to increase trustworthiness (and by extension, facilitate collective action), it is not the only benefit they perceive. Ingroups listed more potential benefits of participation than outgroups and also rated that the ritual is likely to provide benefits for one’s health and financial prospects, getting rid of evil, or purification. If these benefits are not perceived (or are perceived as less likely) by outgroups, the only benefit that outgroups should weigh against ritual costs is the benefit of collective action (or free-riding thereof), which may not by itself tip the cost–benefit trade-off of ritual participation. Indeed, we did not observe any well-estimated difference between ingroups and outgroups in terms of whether ritual participation increases trustworthiness (both groups reported that ritual participation increases trustworthiness more than the hike), a result congruent with our previous finding in Mauritius that Christians trust more Hindus who are signalling their ritual participation compared to non-signalling Hindus (Shaver et al., 2018; but note that this result might be specific to the Mauritian socioecology).

According to our proposition where costs might be understood to guarantee important supernatural benefits, the differential cost–benefit perception may further stabilize extreme rituals by motivating committed members – who see ritual as a means to obtain specific benefits – to engage in even more costly forms of rituals to obtain larger worldly/spiritual benefits (Bioud et al., 2022). Indeed, we previously observed that intense performance of the Kavadi ritual is associated with the reported presence of chronic illness (Xygalatas et al., 2019). By increasing ritual investments and making participation more costly due to the perceived supernaturally induced benefits of ritual participation itself, committed members may inadvertently stabilize collective action by making the cost–benefit trade-off significantly negative for outgroups/uncommitted individuals.

However, it is important to note that the extent to which costs can be raised is likely capped. Too extreme a participation may be considered insincere (Raihani & Power, 2021), and ritual participants need to strike a delicate balance between increasing the costs to obtain larger benefits without seeming like they only participate to obtain personal benefits (which would make them uncooperative). Indeed, our data showed that increasing the intensity of ritual participation was not perceived as leading to larger benefits, seemingly contradicting our theoretical proposition. It is yet again important to consider the Mauritian socioecology. When selecting our stimulus, we strived to balance the costliness of an average performer so that the performance would be easier to identify with. It may be that we inadvertently hit a ceiling of participation intensity for most people, especially because we recruited participants from mid or upper social classes for whom higher physical intensity is seen as unnatural and often frowned upon (Xygalatas et al., 2021). To test this idea, future research might either recruit participants with lower socioeconomic status where costlier performance is more common or manipulate ritual costliness by displaying much less costly ritual participation and test whether the obtained benefits would be considered smaller compared to the current study.

Having argued that some ritual costs may be overestimated by committed members, we should also note that we do not claim that all ritual costs are a subject to these differential estimations. Some signals may indeed be cheaper for committed individuals to produce, especially if they result from long-term investments (Számadó et al., 2023). Take, for instance, the knowledge of sacred texts: reciting from the Vedas itself is almost costless for a person who spent their life memorizing Vedas; such a signal cannot be produced by an outsider. A similar case can be made for a complex ritual performance, which is often learned during early socialization into the religious community. Of course, such signals also require initial costly investments (spending time on memorizing the text), but the performance itself does not need to be costly for committed individuals. In theory, a similar explanation might apply to learning to feel no pain during extreme rituals such that the cost is indeed lower for committed individuals, yet such a modification of the perceptual system needs to assume a complex top-down cognitive process that is difficult to defend with our current empirical knowledge.

Another important clarification relates to the hidden quality that is presumably being signalled. Whereas we framed ritual participation as a costly signal of commitment to collective action, it is possible that such rituals also signal other qualities (Barker et al., 2019; Lang & Kundt, 2024). For example, the strenuous physical effort some individuals exert during the ritual procession may signal their physical aptness and might be appreciated in the marriage market (Van Slyke, 2017). However, our previous research does not support this interpretation, where we saw that Kavadi participants were preferred for marriage by parents of unmarried daughters rather than by the daughters themselves, suggesting it’s a signal of trustworthiness, which is more important for parents than physical prowess (Xygalatas et al., 2022). A likelier possibility is that Kavadi participation, beyond cooperative intent, also signals social status. In our previous research, we observed that social status predicts the form of participation as well as the position in the procession (high-status individuals in front), and there may be additional benefits for some from such signalling (Xygalatas et al., 2021). Indeed, those of high status often do not perform extremely physically costly activities, suggesting that apart from reaping cooperative benefits, they may also reap social status benefits and can afford to decrease the physical cost of participation. Similarly to social status, some participants may also advertise their material wealth by sponsoring mass feasts after the end of the ritual. Although these additional signals complicate the cost–benefit trade-off proposed in this paper, we believe these signals are often complementary to signalling group commitment and, therefore, do not change the overall interpretation of the results.

As with any other empirical research, this study is not without important limitations. First, we cannot be sure whether participants reported the costs and benefits of ritual participation as they perceived them or if this reporting was already part of the signalling game. Although we believe that participants did not have the motivation to signal to research assistants (who were often of different ethnicity/religious tradition), especially because their participation was hypothetical, the assessment of benefit/cost estimation needs to rely on self-reports that can be prone to self-report biases. Nevertheless, the overestimation of financial costs by ingroups is consistent with our previous research (Lang et al., 2025), suggesting that reports in this study might be reliable. Another important limitation is that we asked participants to imagine partaking in the ritual in a specific form – carrying a Kavadi with one piercing for men and carrying a pot with one piercing for women. Although this was necessary to standardize ritual costs across participants (and obtain their differential perception), forcing this form of ritual participation on participants may be unnatural as they might prefer to partake more/less intensely. Nevertheless, as shown in our manipulation check, the modal answer for ingroup participants on how difficult it was to imagine the participation was ‘not at all’, providing solace that the somewhat artificial stimulus was still ecologically valid.

In terms of the cost/benefit assessment, it is possible that non-participation for ingroups and participation for outgroups might bear substantial reputational costs that enter the cost/benefit computation and affect decision-making for whether to partake in the ritual. Although ingroup members may face higher costs of non-participation, annual participation in Kavadi is not necessarily expected of Tamils. For outgroup members, by contrast, participation may carry higher costs, since they might be perceived as suspicious outsiders. At the same time, our data (see Fig. S1 and Section 4.5) suggest that outgroups report Kavadi participation as a stronger trust signal than participation in a hike. These differential costs may therefore contribute to stabilizing costly commitment signals—an effect not assessed in he current study. In our design, these costs would be difficult to compare, but future studies might include direct questions about these effects and include them in the cost/benefit analysis. Moreover, whereas we detected that ingroups perceived the benefits of ritual participation as larger compared to outgroups, participation in the ritual might also be motivated simply by the need to reaffirm cultural identity. That is, some participants might be motivated to partake in the ritual simply to celebrate their community and perpetuate its customs (Xygalatas & Maňo, 2024), and we would not expect outgroups to participate in this ritual because they lack this motivation. Yet, our empirical results show that the differential benefit perception is important, and, moreover, the free-list analysis (Fig. 3) shows that the motivation to reaffirm identity is mentioned by outgroups rather than ingroups. Although outgroups might seem Thaipusam Kavadi as an important part of Tamil identity, they may not necessarily perceive the spiritual and worldly benefits this participation entails. To test this speculation, future studies might compare the cost/benefit estimation in participants with the same cultural identity who either partake or do not partake in the ritual (see also Lang et al., 2025).

Despite these limitations, we believe the current results bring an important update to costly signalling theory of religion by incorporating the differential computation of signalling trade-offs, effectively explaining the persistence of extreme rituals. Although there is no direct link between the hidden quality (cooperative intentions/group commitment) and signalling thereof, this link can be solidified by socialization into perceiving additional benefits of signalling itself, granted by deities to whom the rituals are dedicated. Only properly socialized and committed members would perceive such benefits, motivating them to more costly performance that would not seem to be beneficial enough for outsiders who perceive only the cooperative benefits. This connection between supernatural benefits, costly signals, and social norms may explain why religions are powerful adaptive systems regulating collective action.

## Supporting information

Kundtová Klocová et al. supplementary material Kundtová Klocová et al. supplementary material

## Supplementary material

The supplementary material for this article can be found at https://doi.org/10.1017/ehs.2025.10017.

## Research transparency and reproducibility interest

All our preregistrations, materials, data, and analytical code are accessible at the Open Science Framework repository: https://osf.io/5xcna/.
