# Supplementary material for: Estimated costs and benefits of participation in an extreme ritual in Mauritius
Source: Evol Hum Sci. 2025 Aug 22;7:e29. doi: 10.1017/ehs.2025.10017 (PMC12645328; doi:10.1017/ehs.2025.10017)
Supplement: Kundtová Klocová et al. supplementary material [file S2513843X25100170sup001.docx]

**Estimated costs and benefits of participation in an extreme ritual in Mauritius**

Kundtová Klocová, E.^1^*, Kundt, R.^1^, Puryag, P. V. ^2^, & Lang, M. ^1^*

^1^LEVYNA: Laboratory for the Experimental Research of Religion, Masaryk University, Brno, Czechia

* Corresponding authors: [eva.klocova@mail.muni.cz](mailto:eva.klocova@mail.muni.cz); martinlang@mail.muni.cz

**SUPPLEMENTARY MATERIAL**

## Power analysis

When estimating the necessary sample size, we assumed there would be a negligible difference between in/outgroup in the control condition, but we expected different effects between in/outgroup in the ritual condition. We estimated statistical power needed to detect all four interaction effects of interest (H1-4). In the selection of effect sizes, we balanced expected effects from previous studies (Lang et al., 2025; Xygalatas & Maňo, 2022) with the practical limitations of collecting data in Mauritius: the time and resource constraints that cap the number of potentially survey participants. In time of pre-registration, we aimed to collect 300 participants, but expected to collect 200 participants. Thus, we conducted one power analysis for 300 participants (reported in the main text) and one for 200 participants – reported below.

Using the command *powerSim* from the *simr* package (Green & Macleod, 2016), we conducted 500 Monte Carlo simulations to simulate the expected interaction coefficients for 168 participants (we assumed 15% exclusion based on the predefined criteria) at alpha level 0.05 and statistical power at 80%. Specifically, for H1, we found that the smaller sample size would allow us to detect a difference in slopes between the in- and out-groups of 1 on a 5-point Likert scale. That is, the difference between the control and ritual condition that could be detected was one point higher in in-groups compared to the same difference in out-groups. For H2, 168 participants would allow us to detect with 80% probability a slope difference of 1.5 between the out-group and in-group participants. That is, the difference between the control and ritual condition should be 1.5 higher in in-groups compared to the same difference in out-groups. For H3, because the structure of the data was identical to the structure of the variables used to test H1, we assumed the same effect size. And for H4, the interaction effect detectable with our smaller sample size was 0.9.

## Measures

### Free-list coding

Two independent coders coded data from free-lists. Overarching aggregate categories were constructed based on an iterative analysis of all responses, and the data were subsequently coded into these categories. To assess the level of their initial agreement, Cohen’s Kappa was calculated based on coding responses for 1,347 unique cases. The analysis yielded a Cohen’s Kappa of 0.731, indicating substantial agreement between the coders according to standard interpretation guidelines (Landis & Koch, 1977) and supporting the reliability of the coding scheme and the consistency of coder judgments. Subsequently, all remaining disagreements were solved in a discussion between the two coders, yielding a unified coded dataset.

### Socio-economic status

We pre-registered to calculate the SES variable using factor scores as in Xygalatas et al. (2021), and planned to construct this variable out of four items: profession rating, education level, car ownership, and house ownership. To rate the professions participants listed, we enlisted three independent local raters to evaluate each profession on a 1-5 scale, where 1 – Not at all prestigious, 5 – Highest prestige. To assess consistency among the three independent raters, we calculated the intraclass correlation coefficient (ICC) using a two-way mixed-effects model with absolute agreement (ICC[3,k]). The resulting ICC was 0.511 (95% CI [0.383, 0.616], p < .001), indicating moderate inter-rater agreement (Cicchetti, 1994). Based on this level of consistency, we computed a mean prestige score for each profession by averaging the three individual ratings.

When we aimed to create the SES latent variable as planned, we observed that the four variables were only weakly correlated (Pearson’s r coefficients between 0.10 and 0.37) and, indeed, Cronbach’s alpha revealed that the planned scale was not sufficiently consistent (standardized alpha = 0.47). Thus, in our supplementary analyses, we use the four items separately as covariates.

## Deviations from pre-registration

We originally pre-registered the following hypotheses:

*H1a. According to the costly signaling theory of religion (CSTR), in-group signalers should perceive the cost of the ritual as lower to non-signalers, and this difference should be larger than in the control condition*

*H1b. According to the cost-as-benefits modification of CSTR, in-group signalers should perceive the cost of the ritual as higher to non-signalers, and this difference should be larger than in the control condition*

*H2. The number of listed benefits will be higher in the in-group signalers compared to the out-group non-signalers, and this difference should be larger than in the control condition*

*H3. Ritual should be rated as more efficacious in securing material and spiritual rewards by in-group signalers compared to out-group non-signalers*

*H4. Increased ritual intensity should be associated with increased perceived benefits*

However, hypotheses 3 and 4 are in odds with the planned statistical models pre-registered at OSF. Since our design is inherently build around the GROUP[in vs. out]*TREATMENT[ritual vs. control] interaction, we specified the hypotheses in the main text according to the logic of this interaction (as in H1-H2). Frankly, we do not have a good rationale for the original misstatement of the hypotheses 3 and 4 other than the haste of fieldwork where the pre-registration was quickly written before commencing data collection. Nevertheless, the misstated hypothesis 3 is tested also in the interaction model (and supported). Furthermore, the misstated hypothesis 4 is not really a hypothesis we could statistically assess with our data since there is no predictor in the hypothesis (we asked participants exactly this question, so this hypothesis could be addressed only using descriptive data which are plotted in Figure 2F).

Furthermore, we pre-registered using latent variables of perceived costs and SES, none of which had sufficient internal consistency in our data; thus, we decided to use individual items in the analyses since such models would be unreliable and flawed. Finally, we pre-registered using a binomial model for the test of H2 but used a Gaussian model that fitted better the data.

## Additional analyses

We conducted two types of robustness analyses: first, conducting the same analyses as in the main text on the full sample including participants we excluded based on pre-registered criteria; and, second, the same analyses but including control variables of age, the number of times participants performed Kavadi in the past, and four variables related to socio-economic status (job prestige, education, and car and house ownership. As documented in Tabs. S1, S2, S6, S8, and S10, all the main results were qualitatively unchanged.

Furthermore, we conducted two types of exploratory analyses: dividing our sample between men and women and looking specifically into the Tamil sample on the effects of religiosity on cost and benefit estimation. Regarding the first exploratory analysis, we roughly observed the same effects for both men and women, except for cost estimation: while the effects for women were in the same directions as those for men, the 95% CIs were wider and included zero (see Tabs. S3 and S4). This finding is congruent with our expectations since costly performance is usually driven by men and women rarely engage in the more extreme practices. Moreover, we adapted our stimuli to correspond to this pattern were men carry larger, more ornate kavadis, which might explain why there was a larger potential for men to report costlier performance in terms of the material costs. Conversely, the pressure on men to report no pain would be stronger than on women since women most typically perform with one needle pierced through their cheeks while the number of piercings for men can reach hundreds.

Regarding the interaction between religiosity and treatment in ingroup participants, we found an effect only for the measure of pain, where religiosity was associated with reporting lower pain in kavadi performance, and there was no such effect in the hike condition (see Tab. S5). Religiosity also positively predicted the perceived probability that Kavadi participation bestows benefits, and this positive effect of religiosity was larger than the positive effect of the probability of benefits in the hike, but not statistically different using conventional cutoffs (see Tab. S9). Given the decreased power of our tests (we focused only on the ingroups in the analysis of religiosity), these results indicate that the strongest effects can be found in the culturally most salient domains: reporting no pain for Kavadi performance and reporting larger benefits.

### Robustness tests of H1

| **Table S1. Cumultive link models with excluded participants testing H1 (beta estimates with 95% CIs).** | | | | |
| --- | --- | --- | --- | --- |
|  | | | | |
|  | Time | Money | Pain | Effort |
|  | | | | |
| Affiliation: Outgroup | 0.35 [-0.17, 0.86] | -1 [-1.54, -0.46] | 1.41 [0.85, 1.97] | 1.07 [0.54, 1.62] |
| Condition: Hike | -2.45 [-3.08, -1.84] | -1.85 [-2.45, -1.27] | 1.81 [1.25, 2.38] | 1.29 [0.74, 1.86] |
| Affiliation*Condition | -0.19 [-0.57, 0.19] | -0.5 [-0.88, -0.13] | 0.38 [0.01, 0.75] | 0.03 [-0.34, 0.4] |
| Gender: Woman | 0.29 [-0.49, 1.07] | 0.98 [0.22, 1.73] | -1.1 [-1.86, -0.35] | -0.83 [-1.58, -0.09] |
| Observations | N = 369 | N = 369 | N = 369 | N = 369 |
|  | | | | |
| Note. Reference categories are Ingroup for Affiliation, Kavadi for Condition, and Man for Gender. | | | | |

| **Table S2. Cumultive link models with control variables testing H1 (beta estimates with 95% CIs).** | | | | |
| --- | --- | --- | --- | --- |
|  | | | | |
|  | Time | Money | Pain | Effort |
|  | | | | |
| Affiliation: Outgroup | 0.57 [-0.01, 1.15] | -0.81 [-1.42, -0.21] | 1.33 [0.71, 1.96] | 1.04 [0.44, 1.65] |
| Condition: Hike | -2.39 [-3.02, -1.77] | -1.83 [-2.44, -1.24] | 1.7 [1.12, 2.28] | 1.25 [0.69, 1.83] |
| Affiliation*Condition | 0.19 [-0.61, 1] | 0.9 [0.12, 1.68] | -1.06 [-1.86, -0.27] | -0.87 [-1.65, -0.1] |
| Gender: Woman | -0.19 [-0.59, 0.2] | -0.42 [-0.81, -0.04] | 0.34 [-0.04, 0.73] | -0.04 [-0.42, 0.34] |
| Age | 0.01 [0, 0.03] | -0.01 [-0.03, 0] | -0.01 [-0.03, 0] | -0.01 [-0.03, 0] |
| Kavadi performance | 0.01 [-0.01, 0.03] | 0.02 [0, 0.04] | -0.01 [-0.04, 0.01] | -0.01 [-0.04, 0.01] |
| Job prestige | -0.11 [-0.5, 0.27] | 0.26 [-0.11, 0.64] | 0.13 [-0.26, 0.51] | 0.1 [-0.29, 0.49] |
| Education | 0.03 [-0.11, 0.17] | -0.03 [-0.17, 0.11] | 0.03 [-0.11, 0.16] | -0.11 [-0.25, 0.03] |
| Car | 0.25 [-0.18, 0.68] | 0.19 [-0.23, 0.6] | -0.51 [-0.94, -0.09] | -0.27 [-0.69, 0.14] |
| House | 0.24 [-0.57, 1.08] | 0.79 [-0.04, 1.64] | -0.5 [-1.33, 0.32] | 0.27 [-0.5, 1.04] |
| Observations | N = 360 | N = 360 | N = 360 | N = 360 |
|  | | | | |
| Note. Reference categories are Ingroup for Affiliation, Kavadi for Condition, and Man for Gender. | | | | |

| **Table S3. Cumultive link models for men testing H1 (beta estimates with 95% CIs).** | | | | |
| --- | --- | --- | --- | --- |
|  | | | | |
|  | Time | Money | Pain | Effort |
|  | | | | |
| Affiliation: Outgroup | 0.37 [-0.37, 1.12] | -1.26 [-2.06, -0.48] | 1.97 [1.17, 2.8] | 0.98 [0.21, 1.77] |
| Condition: Hike | -2.5 [-3.42, -1.63] | -2.42 [-3.33, -1.55] | 1.7 [0.89, 2.53] | 0.87 [0.06, 1.68] |
| Affiliation*Condition | 0.46 [-0.65, 1.59] | 1.67 [0.58, 2.78] | -1.64 [-2.75, -0.56] | -0.98 [-2.06, 0.09] |
| Observations | N = 183 | N = 183 | N = 183 | N = 183 |
|  | | | | |
| Note. Reference categories are Ingroup for Affiliation, Kavadi for Condition. | | | | |

| **Table S4. Cumultive link models for women testing H1 (beta estimates with 95% CIs).** | | | | |
| --- | --- | --- | --- | --- |
|  | | | | |
|  | Time | Money | Pain | Effort |
|  | | | | |
| Affiliation: Outgroup | 0.29 [-0.46, 1.04] | -0.76 [-1.56, 0.02] | 1.24 [0.43, 2.06] | 1.31 [0.52, 2.11] |
| Condition: Hike | -2.42 [-3.31, -1.58] | -1.41 [-2.22, -0.62] | 1.95 [1.18, 2.76] | 1.76 [0.98, 2.56] |
| Affiliation*Condition | 0.14 [-0.97, 1.27] | 0.37 [-0.7, 1.46] | -0.87 [-1.97, 0.21] | -0.76 [-1.85, 0.32] |
| Observations | N = 178 | N = 178 | N = 178 | N = 178 |
|  | | | | |
| Note. Reference categories are Ingroup for Affiliation, Kavadi for Condition. | | | | |

| **Table S5. Cumultive link models testing the effects of religiosity in ingroup participants testing H1 (beta estimates with 95% CIs).** | | | | |
| --- | --- | --- | --- | --- |
|  | | | | |
|  | Time | Money | Pain | Effort |
|  | | | | |
| Religiosity | 0.01 [-0.44, 0.45] | 0.01 [-0.47, 0.5] | -0.76 [-1.25, -0.29] | -0.26 [-0.71, 0.18] |
| Condition: Hike | -2.55 [-3.25, -1.89] | -1.66 [-2.28, -1.06] | 2.03 [1.41, 2.68] | 1.28 [0.71, 1.86] |
| Religiosity*Condition | 0.21 [-0.58, 1.05] | -0.12 [-0.88, 0.64] | 0.93 [0.16, 1.7] | 0.26 [-0.44, 0.97] |
| Gender: Woman | -0.05 [-0.63, 0.52] | -0.38 [-0.93, 0.18] | 0.58 [0.01, 1.16] | -0.29 [-0.84, 0.25] |
| Observations | N = 167 | N = 167 | N = 167 | N = 167 |
|  | | | | |
| Note. Reference categories Kavadi for Condition and Man for Gender. | | | | |

### Robustness tests of H2

| **Table S6. OLS models with full sample, control variables, men, and women testing H2 (beta estimates with 95% CIs).** | | | | |
| --- | --- | --- | --- | --- |
|  | | | | |
|  | Full | Controls | Men | Women |
|  | | | | |
| Intercept | 3.51 [3.27 – 3.75] | 2.63 [1.70 – 3.56] | 3.63 [3.28 – 3.98] | 3.43 [3.10 – 3.75] |
| Affiliation: Outgroup | -0.65 [-0.99 – -0.30] | -0.69 [-1.08 – -0.30] | -0.65 [-1.16 – -0.15] | -0.78 [-1.27 – -0.29] |
| Condition: Hike | -0.25 [-0.61 – 0.11] | -0.26 [-0.63 – 0.11] | -0.49 [-1.02 – 0.04] | -0.06 [-0.55 – 0.43] |
| Affiliation*Condition | 0.66 [0.17 – 1.16] | 0.73 [0.23 – 1.24] | 0.71 [-0.00 – 1.43] | 0.78 [0.09 – 1.47] |
| Age |  | 0 [-0.01 – 0.01] |  |  |
| Kavadi performance |  | 0 [-0.01 – 0.02] |  |  |
| Job prestige |  | 0.22 [-0.02 – 0.47] |  |  |
| Education |  | 0.02 [-0.06 – 0.11] |  |  |
| Car |  | -0.06 [-0.33 – 0.21] |  |  |
| House |  | 0.26 [-0.27 – 0.78] |  |  |
| Observations | N = 369 | N = 360 | N = 183 | N = 178 |
|  | | | | |
| Note. Reference categories are Ingroup for Affiliation and Kavadi for Condition. Gender is set as a random factor. | | | | |

| **Table S7. OLS models with religiosity as predictor in ingroup participants testing H2 (beta estimates with 95% CIs).** | |
| --- | --- |
|  | |
|  | Religiosity model |
| Intercept | 3.53 [3.27 – 3.78] |
| Religiosity | 0.03 [-0.29 – 0.36] |
| Condition: Hike | -0.26 [-0.64 – 0.12] |
| Religiosity*Condition | 0.23 [-0.28 – 0.74] |
| Observations | N = 167 |
|  | |
| Note. Reference categories are Ingroup for Affiliation, Kavadi for Condition. Gender is set as a random factor. | |

### Robustness tests of H3

| **Table S8. OLS models with full sample, control variables, men, and women testing H3 (beta estimates with 95% CIs).** | | | | |
| --- | --- | --- | --- | --- |
|  | | | | |
|  | Full | Controls | Men | Women |
|  | | | | |
| Intercept | 0.49 [0.36 – 0.63] | 0.42 [-0.09 – 0.93] | 0.47 [0.29 – 0.66] | 0.52 [0.33 – 0.71] |
| Affiliation: Outgroup | -0.48 [-0.67 – -0.29] | -0.38 [-0.59 – -0.17] | -0.4 [-0.67 – -0.13] | -0.65 [-0.93 – -0.37] |
| Condition: Hike | -0.8 [-1.00 – -0.60] | -0.75 [-0.95 – -0.55] | -0.81 [-1.10 – -0.53] | -0.8 [-1.08 – -0.52] |
| Affiliation*Condition | 0.54 [0.27 – 0.82] | 0.5 [0.23 – 0.78] | 0.53 [0.15 – 0.92] | 0.64 [0.24 – 1.04] |
| Age |  | 0 [-0.01 – 0.01] |  |  |
| Kavadi performance |  | 0.01 [0.00 – 0.02] |  |  |
| Job prestige |  | 0.04 [-0.09 – 0.18] |  |  |
| Education |  | -0.07 [-0.11 – -0.02] |  |  |
| Car |  | 0.01 [-0.14 – 0.16] |  |  |
| House |  | 0.18 [-0.10 – 0.47] |  |  |
| Observations | N = 369 | N = 360 | N = 183 | N = 178 |
|  | | | | |
| Note. Reference categories are Ingroup for Affiliation and Kavadi for Condition. Gender is set as a random factor. | | | | |

| **Table S9. OLS models with religiosity as predictor in ingroup participants testing H3 (beta estimates with 95% CIs).** | |
| --- | --- |
|  | |
|  | Religiosity model |
| Intercept | 0.44 [0.32 – 0.57] |
| Religiosity | 0.37 [0.21 – 0.53] |
| Condition: Hike | -0.75 [-0.93 – -0.56] |
| Religiosity*Condition | -0.17 [-0.42 – 0.08] |
| Observations | N = 167 |
|  | |
| Note. Reference categories are Ingroup for Affiliation, Kavadi for Condition. Gender is set as a random factor. | |

### Robustness tests of H4

| **Table S10. Cimmulative link models with full sample, control variables, men, and women testing H4 (odds ratios with 95% CIs).** | | | | |
| --- | --- | --- | --- | --- |
|  | | | | |
|  | Full | Controls | Men | Women |
|  | | | | |
| Affiliation: Outgroup | 1.28 [0.73 – 2.24] | 1.14 [0.61 – 2.12] | 1.51 [0.68 – 3.35] | 1.07 [0.48 – 2.39] |
| Condition: Hike | 2.38 [1.32 – 4.29] | 2.29 [1.26 – 4.17] | 2.28 [0.96 – 5.44] | 2.46 [1.10 – 5.47] |
| Affiliation*Condition | 0.85 [0.39 – 1.84] | 0.94 [0.43 – 2.08] | 0.77 [0.26 – 2.31] | 0.98 [0.33 – 2.93] |
| Gender | 0.71 [0.48 – 1.04] | 0.7 [0.47 – 1.03] |  |  |
| Age |  | 0.98 [0.96 – 0.99] |  |  |
| Kavadi performance |  | 1.01 [0.99 – 1.03] |  |  |
| Job prestige |  | 1.03 [0.70 – 1.50] |  |  |
| Education |  | 1.04 [0.91 – 1.20] |  |  |
| Car |  | 1.1 [0.73 – 1.68] |  |  |
| House |  | 0.71 [0.32 – 1.57] |  |  |
| Observations | N = 369 | N = 360 | N = 183 | N = 178 |
|  | | | | |
| Note. Reference categories are Ingroup for Affiliation and Kavadi for Condition and Man for Gender. | | | | |

| **Table S11. Cummulative link models with religiosity as predictor in ingroup participants testing H4 (odds ratios with 95% CIs).** | |
| --- | --- |
|  | |
|  | Religiosity model |
| Religiosity | 1.32 [0.77 – 2.24] |
| Condition: Hike | 2.21 [1.24 – 3.94] |
| Religiosity*Condition | 0.84 [0.36 – 1.91] |
| Gender | 0.81 [0.46 – 1.43] |
| Observations | N = 167 |
|  | |
| Note. Reference categories are Ingroup for Affiliation, Kavadi for Condition and Man for Gender. | |

### Supplementary Plots


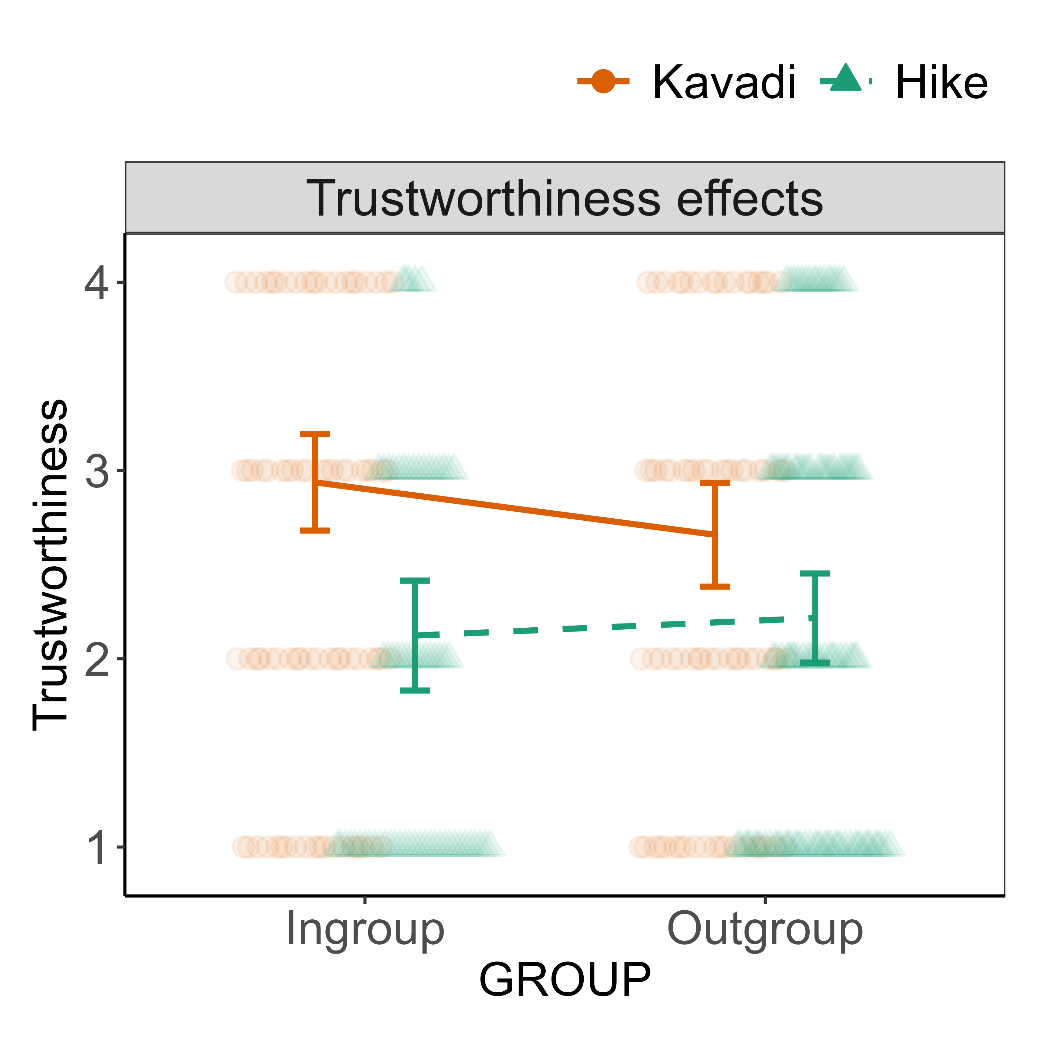


**Figure S1 | Exploratory test of activity participation on trustworthiness.** Error bars are 95% CIs and scatter plots represent raw data.


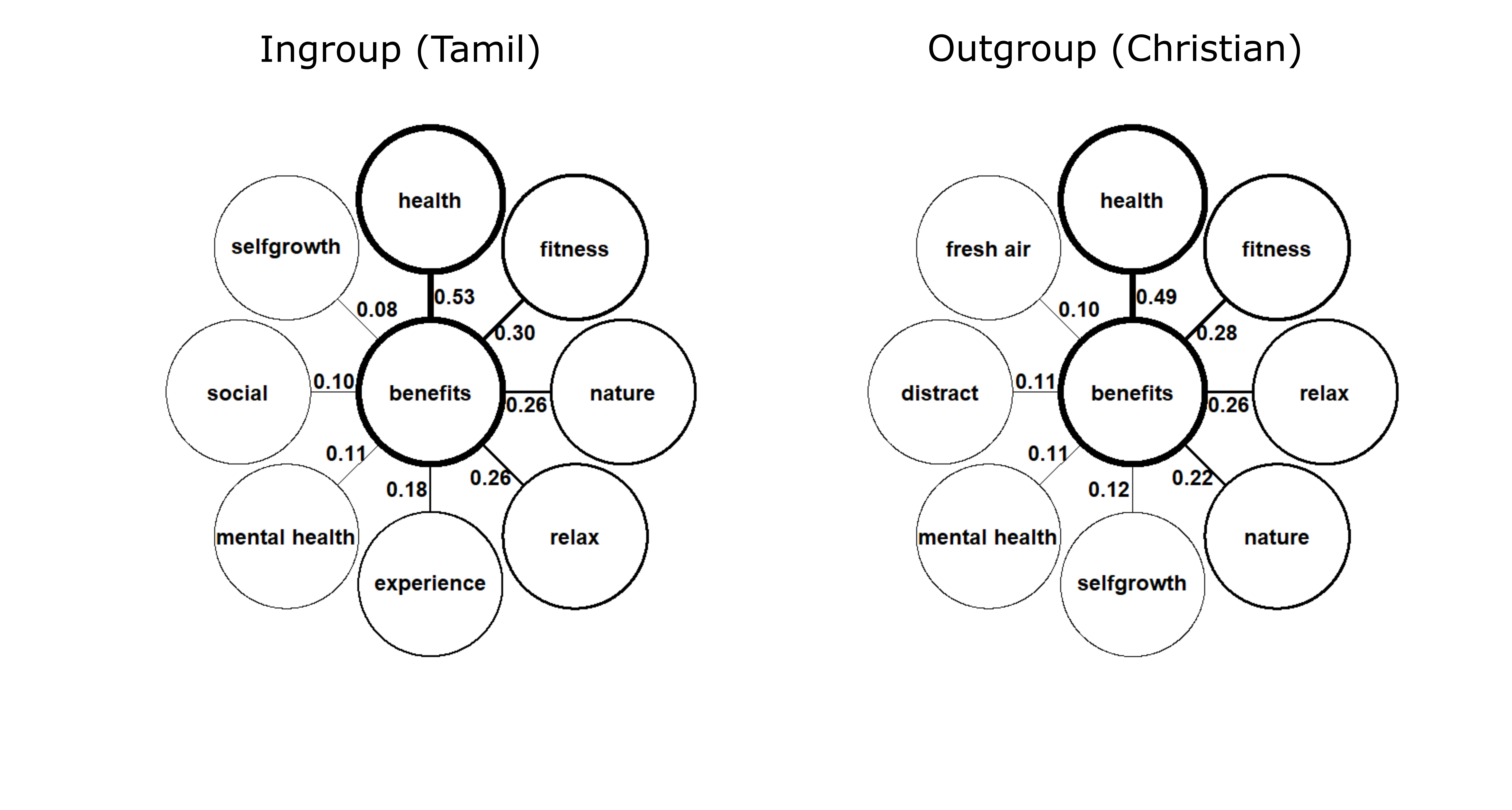


**Figure S2 |** **Flower plots display the most salient benefits of hiking for Tamils and Christians.** The top outer circle shows the most salient item for each group, with descending items listed clockwise. Numbers and weighted border lines indicate the relative salience of each item (Smith's S).
